# Risk Factors for Surgical Site Infections after Paediatric Appendectomies in a Tertiary Care Teaching Hospital in Coastal Karnataka

**DOI:** 10.12688/f1000research.163894.1

**Published:** 2025-06-06

**Authors:** Rajesh Kamath, Anaswara S Kumar, Tarushree Bari, Varshini RJ, Sagarika Kamath, Aswin Sugunan, Sanjeev Chougule,, Siddhartha Sankar Acharya

**Affiliations:** 1 Department of Healthcare and Hospital Management, Prasanna School of Public Health, Manipal Academy of Higher Education, Manipal, Karnataka, India; 2 Directorate of Online Education, Manipal Academy of Higher Education, Manipal, Karnataka, India; 3 Department of Healthcare and Hospital Management, Prasanna School of Public Health,, Manipal Academy of Higher Education, Manipal, Karnataka, India; 4 Department of International Health, Care and Public Health Research Institute—CAPHRI, Faculty of Health, Medicine and Life Sciences, Maastricht University, Maastricht, 6200, Netherlands Antilles; 5 Department of Hospital Administration, Yenepoya University, Mangalore, Karnataka, India; 6 Department of Hospital Administration, JN Medical college, KAHER, KLE University, Belgaum, Karnataka, India; 7 Department of Medical Administration, Tata Memorial Hospital, Mumbai, Maharashtra, 400012, India

**Keywords:** Surgical site infections, appendicitis, paediatrics, appendectomies, risk factors.

## Abstract

**Introduction:**

Surgical site infections (SSIs) occur in 1.3% to 4% of paediatric appendectomies. It is the most common complication after an appendectomy. Surgical complications in paediatric patients increase patient susceptibility to opportunistic infections, falls and drug-related side effects The aim of this study was to identify the modifiable risk factors for SSIs after paediatric appendectomies.

**Methodology:**

Paediatric patients who underwent appendectomies from April 2018 to April 2023 in a tertiary care teaching hospital in coastal Karnataka were included. Clinical characteristics of patients were collected from the medical records of the patients.

**Results:**

A total of 459 paediatric patients underwent appendectomies between 2018 and 2023. 45 (9.8%) developed surgical site infections (SSIs). Chi-squared tests (or Fisher’s exact tests, where appropriate) revealed significant associations (p < 0.05) between SSIs and the following categorical variables: faecal incontinence, surgical technique, wound type, type of appendicitis, postoperative stay and use of invasive devices. Multivariate logistic regression analysis further identified laparotomy (OR = 4.39, 95% CI: 1.31–14.74), presence of invasive devices (OR = 104.4, 95% CI: 5.45–2002.93), longer consultation-to-surgery time (OR = 1.48, 95% CI: 1.17–1.88), and increased surgery duration (OR = 1.02 per minute, 95% CI: 1.01–1.04) as independent risk factors significantly associated with the development of SSIs.

**Discussion:**

The study identified laparotomy, use of drains, delayed surgery, and longer operation time as key risk factors for SSIs after paediatric appendectomies. Despite standard precautions, high SSI rates were observed, likely due to modifiable surgical factors and antimicrobial resistance. The findings highlight the need for optimized surgical practices, timely intervention, and multidisciplinary strategies to reduce SSI rates and improve patient outcomes.

## Introduction

Surgical site infections (SSIs) with an estimated incidence of 2% to 11% are significant health problems that occur after an invasive procedure. ^1^ Their share of all hospital-acquired infections is 19.6% in Europe. ^2^ SSIs affect one-third of surgical patients and are the most common type of hospital acquired infections (HAIs) in low and middle income countries (LMICs). Global estimates for SSIs range from 0.5% to 15%. Studies in India consistently *show higher rates*, ranging from 23% to 38%. ^3^ The combined incidence of SSIs in LMICs is 11.8%. ^4^ In children, SSIs are the most common postoperative complications with an incidence of 2.5% to 5.4%. ^5^ Hospital stays for children with SSIs have been reported to be up to three times longer. SSIs severely impact the health and well-being of affected children and their families. SSIs are associated with significant morbidity, increased antibiotic use, antibiotic-resistant pathogen growth, the possibility of additional surgery and extended hospitalization, all of which increase the burden on healthcare resources. ^6^ Surgical complications in paediatric patients increase patient susceptibility to opportunistic infections, falls and drug-related side effects. The economic costs of SSIs range from an estimated $26,977 to $961,722 due to readmissions and reoperations. ^7^ These infections result in an additional cost of $10 billion in the United States each year, with 4,00,000 additional hospital days. ^8^ SSI prevention and reduction will improve patient outcomes and decrease resource consumption. ^9^

Appendicitis is the most common surgical emergency diagnosis in children. The annual incidence of appendicitis in the United States has been estimated at 86 to 100 cases per 1,00,000 people. The current lifetime risk of appendicitis is 7 to 8 percent. ^10^ Acute appendicitis is the primary cause of abdominal pain in the paediatric population. 1 to 8 percent of children experiencing gastrointestinal pain develop acute appendicitis. ^11^ The highest incidence of appendicitis is in the 10-19 year age group: 23.3 per 10,000 population per year. ^12^ 60,000 to 80,000 paediatric appendectomies are performed annually with a mean cost of $9,000. ^10^ Acute appendicitis is divided into simple and complicated appendicitis (CA). ^13^ Around 33% of appendicitis cases fall under the category of CA which includes gangrenous or perforated appendicitis, phlegmon formation, peri appendicular mass and fecal peritonitis as determined by histology or intraoperative diagnoses. CA is associated with higher morbidity and poorer outcomes such as increased rates of SSIs and intra-abdominal abscesses which result in a delayed return to regular activities and an increase in hospital readmissions. ^14^ Appendiceal perforation is related to greater morbidity and mortality in comparison with non-perforating acute appendicitis. The overall mortality rate for acute appendicitis ranges from 0.3% in non-perforated appendicitis to 6.5% in perforation. ^14^ Delay in diagnosing acute appendicitis and as a result delaying appendectomy can result in severe complications like perforation and peritonitis. The rate of perforation varies from 16% to 40%, with younger age groups experiencing a higher frequency of occurrence (40-57%). ^15^ Appendix perforation causes either diffuse peritonitis or localized appendicular abscesses. Younger children are at a higher risk of experiencing diffuse peritonitis due to their less developed omentum. Older children are better shielded from this condition because they have a well-developed omentum that provides a better defence against it. Escherichia coli, peptostreptococcus, Klebsiella pneumoniae, Bacteroides fragilis and pseudomonas species are the most common aerobic pathogens that cause acute appendicitis. ^11^ The risk of death associated with non-gangrenous acute appendicitis is less than 0.1% and when acute appendicitis progresses to a gangrenous state, the risk of death rises to approximately 0.6%. ^15^

Appendectomy is the most performed treatment for appendicitis and is one of the most common surgeries worldwide, resulting in a significant burden on healthcare systems. ^16^ Surgical site infections are frequently occurring complications following an appendectomy, especially in cases of complicated appendicitis. ^17^ It can occur in up to 9% of appendectomies and is thus the most common complication after appendectomy. ^18^ Appendectomy can be performed in two ways: laparoscopic appendectomy (LA) and open appendectomy (OA), with laparoscopy currently being the most common form of surgery. The laparoscopic approach is associated with reduced postoperative discomfort, a shorter hospital stays, reduced postoperative ileus and superior cosmetic results. ^19^ Since 1894, OA has been the standard treatment for acute appendicitis with proven efficacy and safety. In 1983, Semm carried out the first LA and it has been utilized for more than 30 years now. Both procedures have intraoperative and postoperative complications including SSIs. ^20^ The Center for Disease Control (CDC) classifies SSI as superficial incisional SSI (SSSI), deep incisional SSI and organ/space SSI (OSI). Despite the low mortality rate of acute appendicitis, SSIs, particularly SSSIs and OSIs are the most common complications with incidence rates ranging from 2.5 to 5.4% and 1.3 to 3.0% respectively. ^13, 18^ OSI rates ranging from 7.0% to 15% have been found postoperatively in patients with complicated (gangrenous or perforated) appendicitis. SSI prevalence rates following appendectomy are reported to be 7.2%, 5.9%, 6.2%, and 2.9%, respectively, in studies conducted in Brazil, Sweden, China and the United States. ^19, 21^

Annually more than 80,000 appendectomies are performed on patients younger than 18 years. ^22^ The incidence of acute appendicitis varies across age groups, ranging from 1 to 6 cases per 10,000 children under the ages of 4 to 19 years to 28 cases per 10,000 children under the age of 14 years. When performed laparoscopically, SSIs can occur in 1 to 3 percent of cases and up to 5 percent of laparotomies. Readmissions are required in 5 to 10 percent of patients due to intra-abdominal abscesses and surgical reinterventions are required in less than 1 percent of patients. ^23^ Children are the group of patients who would benefit the most from decreased postoperative complications, earlier mobilization and early discharge from the hospital because the delays can impact their development. ^24^ Diagnosing acute appendicitis in young children is still difficult despite the availability of sophisticated diagnostic imaging techniques because most patients arrive late and have complications. The delay in the diagnosis of acute appendicitis is due to nonspecific presentations, symptom overlap with various prevalent childhood diseases and the challenges associated with conducting abdominal examinations in this particular age cohort. The various orientations of the vermiform appendix cause the nonspecific symptoms of acute appendicitis. If the appendix becomes inflamed in a retrocaecal or subserosal position, there is a lower chance of developing anterior abdominal pain and tenderness. These patients commonly report increased discomfort in the flank or back region as well as symptoms that last longer and an increased rate of perforation. As a result, children are more prone to have problems including perforation and the formation of an abscess. Up to 50 percent of all children with acute appendicitis present with non-specific symptoms. In children aged 2 to 12 years, the rate of misdiagnosis varies from 28 to 57 percent and it approaches 100 percent in those under 2 years of age. ^10^ A large number of differential diagnoses complicate the diagnosis of acute appendicitis. Acute appendicitis has hence been considered as the “chameleon of surgery.” Acute gastroenteritis, urinary tract infections, testicular torsion, nephrolithiasis, intussusception, blunt abdominal trauma, pelvic inflammatory disease, orchitis, constipation, cholecystitis, obstructed hernia are among the differential diagnoses in the paediatric age group. ^25^^,^^26^

The core of treatment for SSI prevention following appendectomy for complicated appendicitis is ongoing antibiotic therapy. ^27^ In 2017, the Surgical Infection Society (SIS) and the Infectious Disease Society of America (IDSA) jointly released guidelines for managing community acquired intra-abdominal infections specifically aimed at patients aged over one month. The authors recommend that low-risk patients should receive a treatment regimen that includes cefotaxime/ceftriaxone along with metronidazole or ertapenem. Piperacillin-tazobactam, imipenem or meropenem are to be administered to high-risk patients. A five-day course of antibiotics is also advised. It has been shown that oral antibiotics taken at home following hospital discharge are as effective as intravenous (IV) antibiotics in preventing SSIs. Some studies suggest that the use of oral antibiotics following discharge at home may be a preferable strategy compared to not administering antibiotics for the prevention of SSIs. ^26^ The aim of this study was to identify the modifiable risk factors for SSIs in paediatric patients undergoing appendectomies in a tertiary care teaching hospital in coastal Karnataka, South India.

## Methods

### Study setting

A retrospective, single centre, exploratory study was conducted for 6 months from September 2024 to February 2025 in a tertiary care teaching hospital in coastal Karnataka. The hospital is a premier healthcare facility with 2000 plus beds dedicated to providing tertiary medical services to a wide range of patients. The study was conducted to identify the modifiable risk factors associated with the occurrence of surgical site infection among paediatric patients who had undergone an appendectomy. The data was collected for a period of 4 weeks from the Medical records department of the hospital using a developed and validated proforma.

### Study design

The study was a retrospective exploratory single centre study.

### Departments involved

Medical records department.

### Inclusion criteria

Patients between the ages of 1 and 18 years who underwent appendectomy between April 2018 and April 2023 and were readmitted to the hospital with purulent drainage, abscess, pain, redness, heat and other symptoms from the wound within 30 days of surgery.

### Exclusion criteria

Patients with acquired or congenital immunodeficiencies, autoimmune disorders and cancer who were predisposed to infections.

### Sample size

The study included a total of 459 patients who had undergone appendectomy from April 2018 to April 2023. This study did not employ a predetermined sample size calculation. All eligible patients within the timeframe were included to maximize the available data and ensure comprehensive representation.

### Statistical methods

Descriptive statistics, inferential statistics and logistic regression analysis were used for analysis. Qualitative variables were depicted using relative and absolute frequencies. The Shapiro-wilk test was done to check the normality of quantitative variables which were found to be not normally distributed. After examining the distributions, quantitative variables were explained using appropriate measures of central tendency and dispersion. The Chi-squared test was used to assess the independence of qualitative variables. In cells with values less than 5, the Fishers exact test was used. Given the study design, which involved the comparison of cases (patients with SSI) and controls (patients without SSI), the odds ratio was chosen as the appropriate measure of association. The odds ratio was calculated to assess the strength of association between potential risk factors and the likelihood of developing SSI. A 95% confidence interval logistic regression model was used to calculate the odds ratio. The analysis was carried out with Jamovi version 2.3.18 and the results were formulated.

### Tools used

A Validated proforma was used. The validated proforma is available in the data repository mentioned in the data availability statement below.

### Study variables

The study included sociodemographic parameters such as gender and age, clinical metrics such as height and weight to determine the Body Mass Index (BMI) and nutritional status, procedure-specific factors such as the aseptic solution used, surgical technique/s deployed, type of wound, surgical duration, classification of appendicitis, postoperative accommodation, antibiotic prophylaxis, presurgical antisepsis and the use of invasive apparatuses such as drains. All the variables were found in the medical records of patients who met the inclusion criteria. These variables were collected for both cases (patients who developed SSIs) and controls (patients who did not develop SSIs).

### Ethical approval

The study was approved by the Institutional Ethics Committee-2 (Student Research) of Kasturba Medical College and Kasturba Hospital, Manipal. Ethical approval was granted on 26th October 2023, under approval number IEC2: 573/2023. As the study is retrospective in nature, patient consent was not required. Data were collected from the Medical Records Department after obtaining ethical approval. Confidentiality of information was strictly maintained, and no identifiable images or personal data of individuals were used in the study.

## Results

Table 1 presents a total of 459 paediatric patients who underwent appendectomies between April 2018 and April 2023 were included in this study. 45 (9.8%) patients developed SSIs, while 414 (90.2%) did not and served as the control group.

**Table 1.** Clinical characteristics of paediatric patients who underwent appendectomy. ^41^

Variable	Total (N = 459)	Cases (N = 45)	Controls (N = 414)	p-value
Age (Median, IQR)	11 (6)	10 (7)	11 (6)	-
Male (%)	265 (57.7)	17 (37.8)	177 (42.8)	0.5
Female (%)	194 (42.3)	28 (62.2)	237 (57.2)	0.5
BMI (Median, IQR)	16.0 (4.0)	15.7 (3.8)	16.0 (4.0)	-
Underweight (%)	130 (28.3)	13 (28.9)	117 (28.3)	0.5 *
Faecal incontinence (%)	32 (7.0)	8 (17.8)	24 (5.8)	0.003
Type of wound (%)				
- Contaminated	316 (68.8)	2 (4.4)	314 (75.8)	<0.001 *
- Dirty	143 (31.2)	43 (95.6)	100 (24.2)	
Type of Appendicitis (%)				
- Early	141 (30.7)	0 (0.0)	141 (34.1)	<0.001 *
- Suppurative	167 (36.4)	2 (4.4)	165 (39.9)	
- Gangrenous	10 (2.2)	2 (4.4)	8 (1.9)	
- Perforated	141 (30.7)	41 (91.1)	100 (24.2)	
Invasive devices (%)	149 (32.4)	44 (97.8)	105 (25.4)	<0.001
Post-operative stay (%)				
- Hospitalization	455 (99.1)	41 (91.1)	414 (100)	<0.001 *
- Paediatric ICU	4 (0.9)	4 (8.9)	0 (0.0)	

The median age of patients in the SSI group was 10 years (IQR: 7) while in the control group it was 11 years (IQR: 6). Males constituted 57.7% (n = 265) of the total sample, but gender distribution was not significantly associated with the development of SSIs (p = 0.5).

* Fisher's exact test was used for variables where cell count was below 5.

### Demographic and clinical characteristics

**Figure 1.**
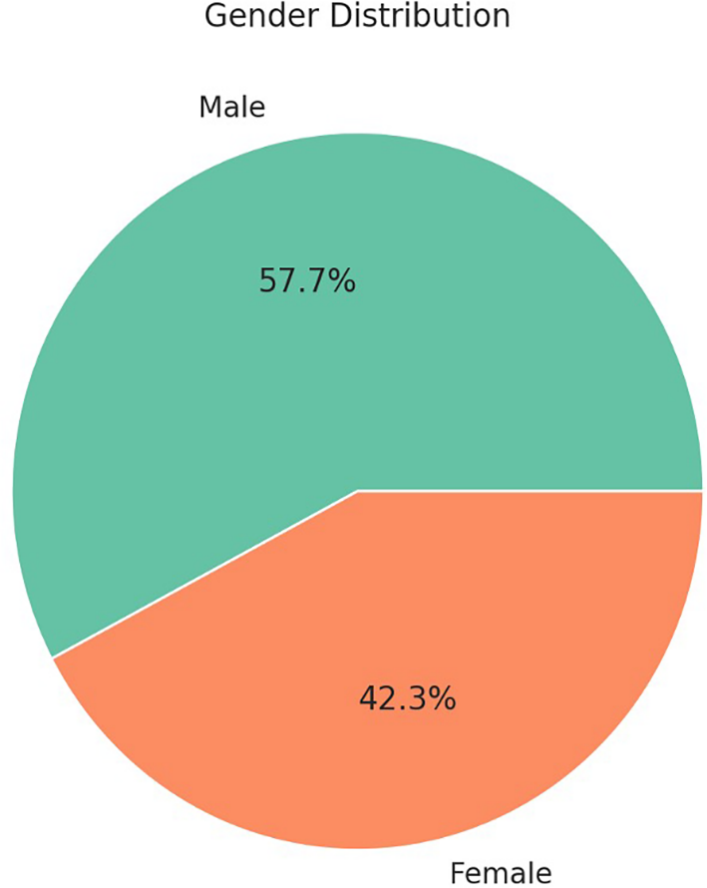
Gender distribution of paediatric patients who underwent appendectomy. ^44^ Figure illustrates the gender distribution of paediatric patients who underwent appendectomy ^41^

Table 1 presents the median age of patients in the SSI group was 10 years (IQR: 7) while in the control group it was 11 years (IQR: 6). Figure 1 visually depicts males constituted 57.7% (n = 265) of the total sample, but gender distribution was not significantly associated with the development of SSIs (p = 0.5), as shown in Table 1.

Nutritional status also showed no significant difference between cases and controls.

### Surgical and infection-related factors

**Figure 2.**
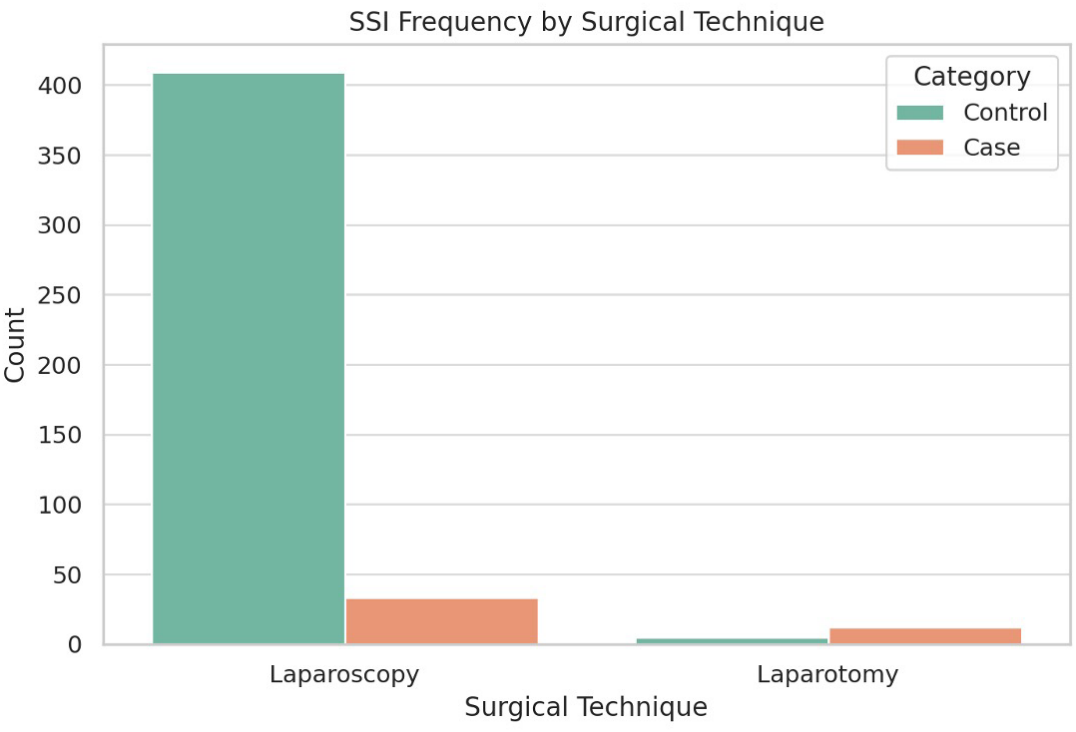
Distribution of surgical techniques (Laparoscopy vs Laparotomy) among cases and controls. ^44^ Figure shows the distribution of surgical techniques—laparoscopy and laparotomy—among paediatric appendectomy patients, highlighting a higher proportion of laparotomy procedures among cases with surgical site infections compared to controls. ^41^

**Figure 3.**
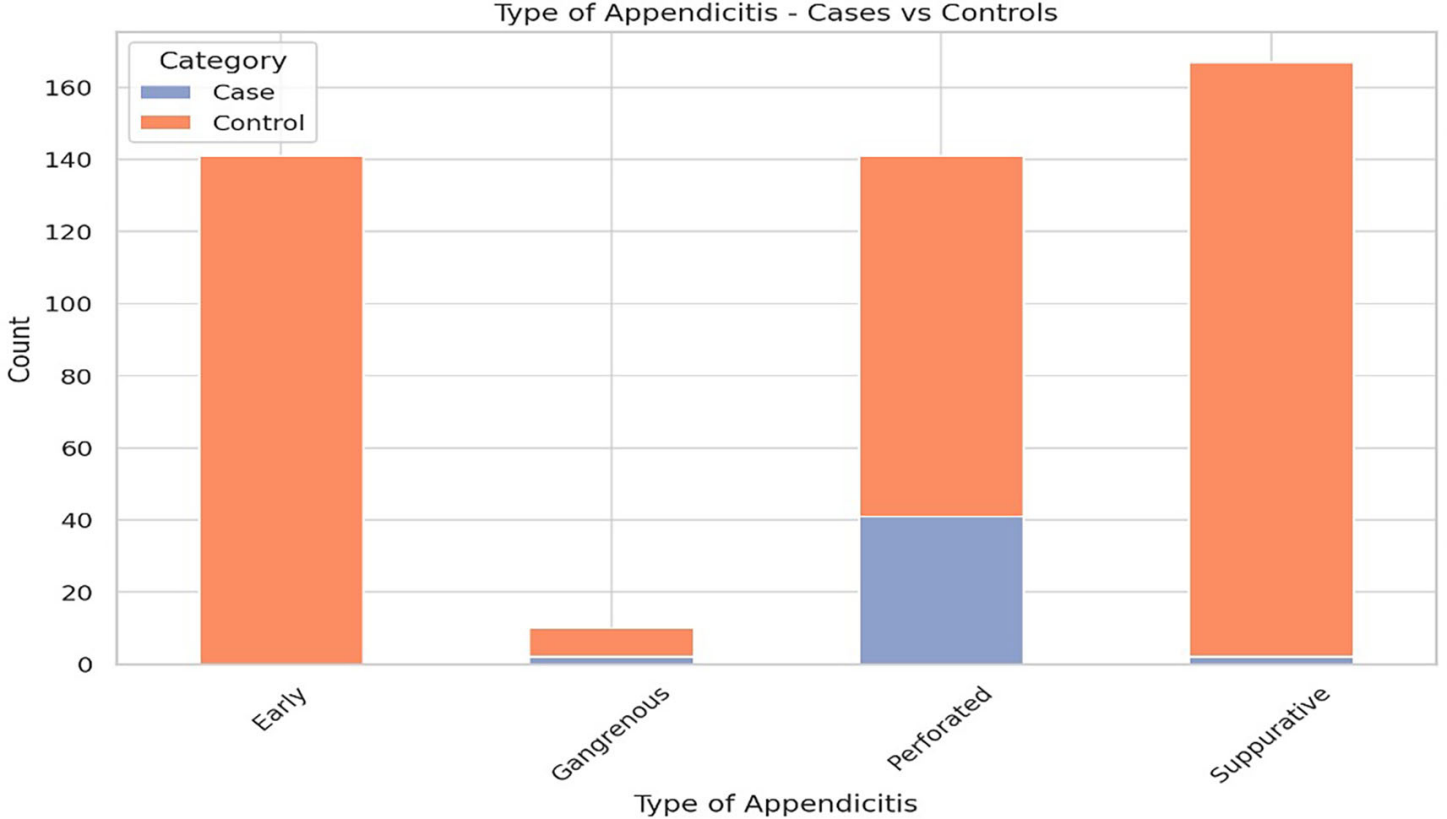
Distribution of type of appendicitis (early, suppurative, gangrenous, perforated) among cases and controls. ^44^ Figure displays the distribution of appendicitis types—early, suppurative, gangrenous, and perforated—among paediatric appendectomy patients, revealing a predominance of perforated appendicitis among cases with surgical site infections compared to controls. ^41^

**Figure 4.**
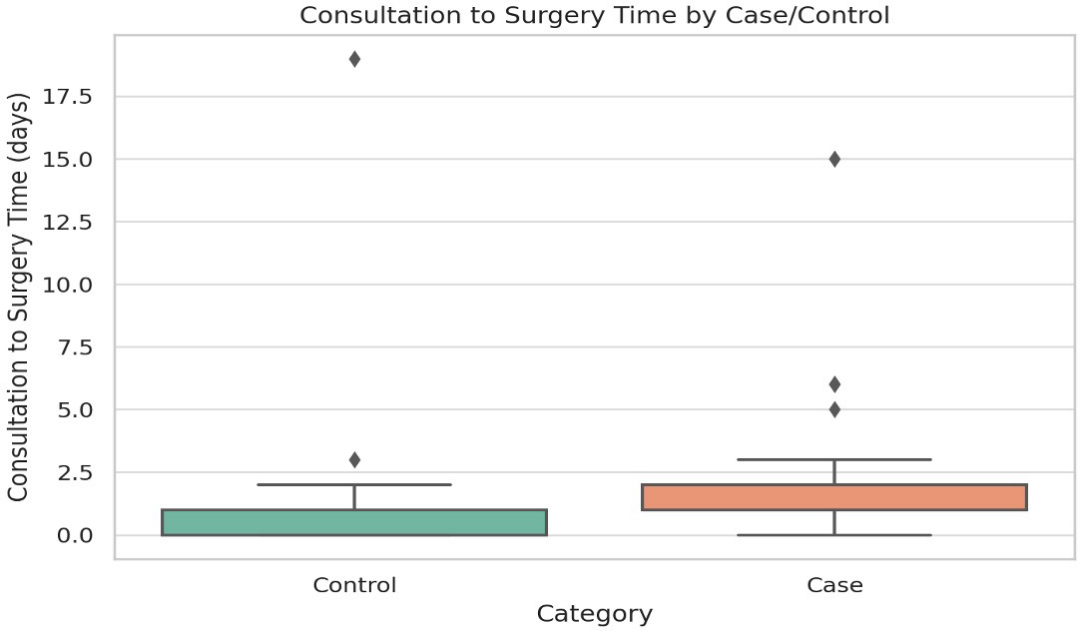
Comparison of initial consultation-to-surgery time between cases and controls. ^44^ Figure compares the time from initial consultation to surgery between cases and controls, showing that patients who developed surgical site infections experienced a significantly longer delay before undergoing surgery. ^41^

**Figure 5.**
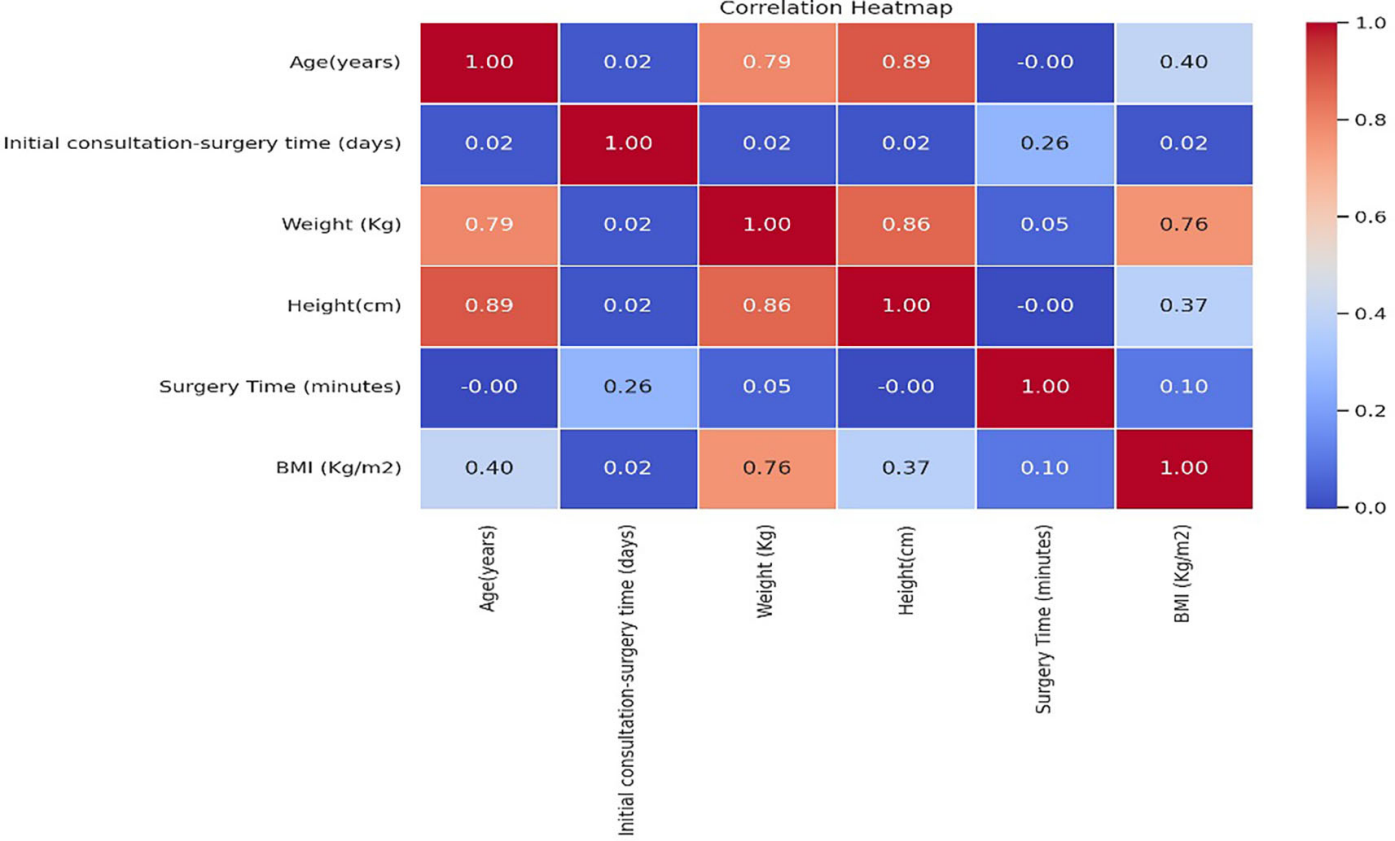
Correlation heatmap showing relationships between numerical variables among paediatric appendectomy patients. ^44^ Figure presents a correlation heatmap depicting the relationships between numerical variables among paediatric appendectomy patients, highlighting significant positive correlations between consultation-to-surgery time, surgery duration, and the likelihood of developing surgical site infections. ^41^

• **Surgical technique:** 26.7% (n = 12) of the SSI cases underwent laparotomy, while only 1.2% (n = 5) in the control group had laparotomies (p < 0.001). Patients undergoing laparotomy had significantly higher odds of developing SSIs (OR = 4.39, 95% CI: 1.31-14.74, p = 0.01) as shown in Table 2. The distribution of surgical techniques is illustrated in Figure 2.
• **Type of wound:** A strong association was observed between wound classification and SSI occurrence. 95.6% (n = 43) of patients with SSIs had dirty wounds, whereas 75.8% (n = 314) in the control group had contaminated wounds (p < 0.001), as presented in Table 1.
• **Type of appendicitis:** Among patients who developed SSIs, 91.1% (n = 41) had perforated appendicitis, whereas only 24.2% (n = 100) in the control group had this condition (p < 0.001), as depicted in Figure 3 and presented in Table 1.
• **Use of invasive devices:** 97.8% (n = 44) of SSI cases had invasive devices (drains) placed postoperatively compared to 25.4% (n = 105) in the control group (p < 0.001). The odds of developing SSIs were extremely high for patients with invasive devices (OR = 104.4, 95% CI: 5.45-2002.9, p = 0.002), as indicated in Table 2.

• **Initial consultation to surgery time:** The median time was 1 day for SSI cases and 0 days for controls (p = 0.001), as depicted in Figure 4. A delay in surgical intervention was significantly associated with increased risk (OR = 1.48, 95% CI: 1.17–1.88, p = 0.001), as presented in Table 2.
• **Surgery duration:** The median time was 120 minutes for cases and 60 minutes for controls (p < 0.001). Each minute increase in surgery time was associated with a higher risk of SSIs (OR = 1.02, 95% CI: 1.01–1.03), as presented in Table 2. This correlation is depicted in Figure 5.
  • The heatmap visualizes the strength and direction of correlations between different clinical and surgical variables, helping identify key risk factors associated with SSIs.
  • The median consultation-to-surgery time was 1 day in SSI cases and 0 days in controls (p = 0.001), suggesting that delays in surgery contribute to increased SSI risk.
  • The median surgery duration was 120 minutes in SSI cases and 60 minutes in controls (p < 0.001), reinforcing that longer surgical times significantly increase the risk of SSIs.

**Table 2.** Risk factors for SSIs among paediatric appendectomy patients. ^41^

Risk Factor	Coefficient	OR (95% CI)	p-value
Surgical technique (Laparotomy)	1.4	4.39 (1.31-14.74)	0.01
Invasive devices (Drains)	4.6	104.4 (5.45-2002.9)	0.002
Initial consultation-to-surgery time	0.3	1.48 (1.17-1.88)	0.001
Surgery time (per minute increase)	0.02	1.02 (1.01-1.03)	<0.001

### Microbiological findings

Table 3 presents the distribution of microbial pathogens among SSI cases. Escherichia coli (E. coli) was the most common organism, accounting for 88.9% of infections followed by Pseudomonas aeruginosa, accounting for 4.4% of infections.

**Table 3.** Distribution of microbial pathogens among SSI cases. ^41^

Microbial Pathogen	Cases (N = 45)	Percentage (%)
Escherichia coli (E. coli)	40	88.9
Pseudomonas aeruginosa	2	4.4
Streptococcus aureus	1	2.2
Streptococcus constellatus	1	2.2
Enterococcus species	1	2.2

## Discussion

The findings of this study provide insights into modifiable risk factors associated with SSIs after paediatric appendectomies in a tertiary care teaching hospital in coastal Karnataka. The study showed that SSIs occurred in 9.8% (n = 45) of patients who underwent appendectomies from April 2018 to April 2023. This is higher than the incidence rates of SSIs found in other studies conducted in Saudi Arabia (7.2%) ^28^ and Netherlands (6.6%). ^13^ A systematic literature review found that the incidence of SSIs increased as a country’s income level decreased. Out of the total, 5.9% of the patients had OSI and 3.9% of the patients had SSSI. The rate of OSI is higher in this study compared to other studies conducted globally (1.6-4.6%). ^13, 19^ In this study, the surgical technique (laparotomy) was found to be a risk factor for SSIs. (OR = 4.39, 95% CI = 1.31-14.74106, p = 0.017). A significant relationship between the surgical technique (laparoscopy or laparotomy) and the risk of SSI (p > 0.001, p = 0.0001), with laparotomies having a higher risk of postoperative wound infection. ^28, 29^ A meta-analysis reported that the incidence of SSIs was 3.29% in patients who underwent laparoscopic appendectomies whereas it was 7.78% in patients who underwent laparotomies. ^30^ This might be due to the larger incision size associated with laparotomies as well as the shorter hospitalization time of the laparoscopic approach.

This study found that the development of SSIs is associated with invasive devices such as drains (OR = 104.46, 95% CI = 5.45-2002.93390, p = 0.002). It was discovered that patients with drains had an abscess rate of 7.4% following an appendectomy, while patients without drains had an abscess rate of 2.1% (p = 0.027). ^31^ Similarly, a study identified that the rate of SSI was 51.1% in patients with drains while it was 25.4% in patients with no drains. ^32^ The study found that the initial consultation to surgery time (days) is a risk factor for SSIs (OR = 1.48, 95% CI =1.17-1.88127, p = 0.001). A study discovered a significant and independent association between the incidence of SSI and delaying appendectomy for longer than six hours (OR (95% CI), 1.54 (1.01-2.34); P = 0.04). Patients with nonperforated appendicitis were found to be especially vulnerable to appendectomy delays. In this group of patients, a 6-hour delay in appendectomy increased the risk of SSIs from 1.9% to 3.3%. ^33^ In contrast, the findings of a study for children with acute appendicitis showed that the delay in surgery was not an independent risk factor for post-operative complications. They found no evidence of a higher rate of perforations because of surgical delay. ^34, 35^

Surgery time was also identified as a risk factor for SSIs in this study (OR = 1.02, CI = 1.01-1.03755, p < 0.001). It has been discovered that the duration of surgery is a significant factor associated with the incidence of SSI (p = 0.0001). ^28^ Another study discovered that operative times greater than 75 minutes were significantly associated with superficial SSIs. ^36^ This could be due to prolonged exposure to contaminants, increased tissue trauma, compromised blood supply and a weakened immune response of the paediatric patients. Weight and BMI of the patients were also found to be risk factors for SSIs in different studies. A study identified that having a low BMI is a risk factor for SSIs after a laparoscopic appendectomy. ^37^ Another study that BMI greater than or equal to 27 kg/m ^2^ was a significant patient related risk factor in developing SSIs post-surgery. ^38^ But in contrast to the previous studies, this study did not identify BMI as a risk factor for SSIs. Several studies have found a significant association between complicated appendicitis and the development of SSIs. In the context of this study complicated appendicitis was not found to be a risk factor for SSIs. However, in this study it was observed that 91.1% of the patients who developed SSIs had perforated appendicitis. Complex appendicitis is identified as a risk factor for developing SSIs after appendectomies both in the paediatric and adult populations. ^13, 39^ Another study found that the rate of SSI and intraabdominal abscess development was 10.7% and 5.8% respectively in patients who had complicated appendicitis. ^40^

Preoperative skin antisepsis was done with povidone iodine for all the patients included in this study. A study by Lee I et al. discovered that patients who received preoperative skin antisepsis with chlorhexidine had a 36% lower rate of SSIs than patients who received povidone iodine. ^41^ Another study found that compared to povidone-iodine, chlorhexidine reduced the risk of SSI by 41%. ^42^ The usage of povidone iodine as the primary antiseptic agent in this study might have contributed to the higher SSI incidence rates. Antibiotic prophylaxis was administered 60 minutes prior to surgery as per the institutional protocol to all the patients included in this study. A meta-analysis found that antibiotic prophylaxis is an effective intervention in preventing SSIs after multiple surgical procedures including simple and complex appendectomies. ^43^ Despite the administration of prophylactic antibiotics, a significant number of patients developed SSI in this study. This indicates that other factors such as the spectrum of antibiotic coverage, the timing of administration, dosage and other patient factors impact the efficacy of prophylactic antibiotics. SSIs are primarily caused by microorganisms resistant to commonly used antimicrobials and can be multidrug-resistant. Antibiotic resistance can occur in more than 50% of SSIs. The micro-organisms isolated from the cases in this study were E.coli (n = 40, 88.9%), Pseudomonas aeruginosa (n = 2, 4.4%), Streptococcus aureus (n = 1, 2.2%), Streptococcus constellatus (n = 1, 2.2%) and Entercoccus species (n=1, 2.2%). It is concerning because E. coli has the highest proportion of antibiotic resistance and is resistant to oxacillin/methicillin in 43% of cases and fluoroquinolones in 25% of cases. ^19^ This could have resulted in the significant rate of SSIs in this study even after the administration of prophylactic antibiotics.

The retrospective single center study investigating risk factors for SSIs following paediatric appendectomies in a tertiary care hospital revealed significant findings. Surgical technique, particularly laparotomy emerged as a risk factor for SSIs, suggesting a potential area for targeted intervention. This underscores the importance of exploring alternative surgical approaches, such as laparoscopic appendectomy which have been associated with reduced SSI rates in previous studies. The presence of invasive devices such as drains were identified as a significant risk factor for SSIs, highlighting the need for judicious use and vigilant monitoring of these devices postoperatively. The study also identified prolonged initial consultation-surgery time and surgery time as modifiable risk factors associated with increased SSI incidence. This emphasizes the importance of timely intervention and efficient surgical scheduling to minimize the duration of postoperative and intraoperative periods thereby reducing the risk of bacterial colonization and subsequent infections. These findings collectively underscore the importance of optimizing surgical practices and streamlining patient care pathways to mitigate the risk of SSIs in paediatric appendectomy cases. Multidisciplinary collaboration involving surgeons, anesthesiologists, nurses and infection control specialists is essential to develop comprehensive strategies aimed at reducing SSI rates and improving patient safety.

## Ethical approval

The study was approved by the Institutional Ethics Committee-2 (Student Research) of Kasturba Medical College and Kasturba Hospital, Manipal. Ethical approval was granted on 26th October 2023, under approval number IEC2: 573/2023. As the study is retrospective in nature, patient consent was not required. Data were collected from the Medical Records Department after obtaining ethical approval. Confidentiality of information was strictly maintained, and no identifiable images or personal data of individuals were used in the study.

## Data Availability

Figshare: Risk Factors for Surgical Site Infections after Paediatric Appendectomies in a Tertiary Care Teaching Hospital in Coastal Karnataka. Doi: https://doi.org/10.6084/m9.figshare.28731197 ^44^ The project contains the following underlying data: SSI.xlsx (The data set includes categorical variables such as sex, presence of fecal and urinary incontinence, type of skin asepsis used, surgical technique (laparoscopy or laparotomy), type of wound (contaminated or dirty), and clinical type of appendicitis (suppurative, early, or perforated). It also includes whether postoperative hospitalization and invasive devices were involved. Additionally, it includes numerical data on patient age and the time interval (in days) between initial consultation and surgery.) Data are available under the terms of the Creative Commons Attribution 4.0 International license (CC-BY 4.0).
