# Supplementary material for: Risk Factors for Surgical Site Infections after Paediatric Appendectomies in a Tertiary Care Teaching Hospital in Coastal Karnataka
Source: F1000Res. 2025 Jun 6;14:565. [Version 1] doi: 10.12688/f1000research.163894.1 (PMC12800604; doi:10.12688/f1000research.163894.1)
Supplement: Peer review history

# Reviewer report: Reviewer response for version 1

**DOI:** 10.5256/f1000research.180314.r448217

**Published:** 2026-01-13

**Authors:** Prashant Tubachi

**Affiliations:** 1 Shri Dharmasthala Manjunatheshwara University, Manjushree Nagar, Karnataka, India

This is very common surgery performed in surgical wards. This study validates the parameters for surgical site infection. This study will play important role in counselling patients and parents about the surgery. Post op surgical site infections can be minimised .Need of laparoscopy and expertise in this technique is promoted. Need of proper antibiotics in complicated cases of appendicitis is validated.

Is the work clearly and accurately presented and does it cite the current literature?

Yes

If applicable, is the statistical analysis and its interpretation appropriate?

I cannot comment. A qualified statistician is required.

Are all the source data underlying the results available to ensure full reproducibility?

Yes

Is the study design appropriate and is the work technically sound?

Yes

Are the conclusions drawn adequately supported by the results?

Yes

Are sufficient details of methods and analysis provided to allow replication by others?

Yes

Reviewer Expertise:

My area of research ,minimal invasive surgery,breast and thyroid surgeries.Diabetic foot ulcers.Ethics in surgery.

I confirm that I have read this submission and believe that I have an appropriate level of expertise to confirm that it is of an acceptable scientific standard.

# Reviewer report: Reviewer response for version 1

**DOI:** 10.5256/f1000research.180314.r393030

**Published:** 2025-07-02

**Authors:** Kumar hari rajah

**Affiliations:** 1 Taylor's University, Subang Jaya, Malaysia

This is a retrospective study on the risk factors for surgical site infection after pediatric appendectomies in a tertiary care teaching hospital.The abstract is well written but the conclusions are not present.The key words are relevant to this topic.The introduction section is well written and highlights the problem of surgical site infections after appendectomies in the pediatric population.The methodology section with its inclusion and exclusion criteria are well written.The results section with its tables and images are accurate.The discussion section is well written and highlights some of the factors that can lead to increased risk of surgical site infection like the type of operation,onset of symptoms.There is however no conclusion section.The references are good and accurate.

Is the work clearly and accurately presented and does it cite the current literature?

Yes

If applicable, is the statistical analysis and its interpretation appropriate?

Yes

Are all the source data underlying the results available to ensure full reproducibility?

Yes

Is the study design appropriate and is the work technically sound?

Yes

Are the conclusions drawn adequately supported by the results?

Yes

Are sufficient details of methods and analysis provided to allow replication by others?

Yes

Reviewer Expertise:

My area of expertise is in general surgery and gastroenterology

I confirm that I have read this submission and believe that I have an appropriate level of expertise to confirm that it is of an acceptable scientific standard.
